# Binge Drinking and Blood Pressure: Cross-Sectional Results of the HAPIEE Study

**DOI:** 10.1371/journal.pone.0065856

**Published:** 2013-06-07

**Authors:** Andrzej Pajak, Krystyna Szafraniec, Ruzena Kubinova, Sofia Malyutina, Anne Peasey, Hynek Pikhart, Yuri Nikitin, Michael Marmot, Martin Bobak

**Affiliations:** 1 Institute of Public Health, Jagiellonian University Medical College, Krakow, Poland; 2 Centre for Health Monitoring, National Institute of Public Health, Prague, Czech Republic; 3 Institute of Internal Medicine, Siberian Branch of Russian Academy of Medical Sciences, Novosibirsk, Russia; 4 Department of Epidemiology and Public Health, University College London, London, United Kingdom; Fundación para la Prevención y el Control de las Enfermedades Crónicas No Transmisibles en América Latina (FunPRECAL), Argentina

## Abstract

**Objectives:**

To investigate whether binge drinking pattern influences blood pressure independently from drinking volume or whether it modifies the effect of volume of drinking.

**Methods:**

We used cross-sectional data from population samples of 7559 men and 7471 women aged 45–69 years in 2002-05, not on antihypertensive medication, from Russia, Poland and Czech Republic. Annual alcohol intake, drinking frequency and binge drinking (≥100 g in men and ≥60 g in women in one session at least once a month) were estimated from graduated frequency questionnaire. Blood pressure was analysed as continuous variables (systolic and diastolic pressure) and a binary outcome (≥140/90 mm Hg).

**Results:**

In men, annual alcohol intake and drinking frequency were strongly associated with blood pressure. The odds ratio of high blood pressure for binge drinking in men was 1.62 (95% CI 1.45–1.82) after controlling for age, country, body mass index, education and smoking; additional adjustment for annual alcohol intake reduced it to 1.20 (1.03–1.39). In women, the fully adjusted odds ratio of high blood pressure for binge drinking was 1.31 (1.05–1.63). Binge drinking did not modify the effect of annual alcohol intake. Consuming alcohol as wine, beer or spirits had similar effects.

**Conclusions:**

The results suggest that the independent long-term effect of binge drinking was modest, that binge drinking did not modify the effect of alcohol intake, and that different alcoholic beverages had similar effects on blood pressure.

## Introduction

It has been long known that drinking alcohol is related to the risk of hypertension. Most studies reported a J-shaped (or a threshold based) association between alcohol and hypertension [1] or a linear relationship, with even low intakes increasing blood pressure [2]–[6]. Randomised trials and Mendelian randomization studies are also consistent with linear effects of alcohol on blood pressure and hypertension [7], [8]. However, as the vast majority of studies have been conducted in western populations, consisting from predominantly regular and moderate drinkers, most of the evidence on alcohol and blood pressure relates to regular moderate drinking pattern.

It has been proposed that in addition to the volume of drinking, the pattern of drinking is also important for cardiovascular risk [9]–[11]. There is emerging evidence that episodic consumption of high amounts of alcohol (binge drinking) is associated with high risk of coronary heart disease [12], stroke [13] and metabolic syndrome [14]. So far, however, there is surprisingly little evidence about the effects of binge drinking on blood pressure. A comparison of blood pressure variation by day of the week in France and Northern Ireland suggested that binge drinking (common in Northern Ireland) may increase blood pressure [15] but individual-level studies produced inconsistent results [16]–[20], perhaps because many of them were relatively small and some examined acute effects of drinking. In addition to binge drinking, beverage preference has also been seen as contributing to drinking pattern [21], and it was suggested that different alcoholic beverages may have a differential effect on cardiovascular system and diseases [22].

Given the relatively high frequency of binge drinking in eastern Europe, and especially in Russia, with approximately one third of Russian men reporting binge drinking [23], [24] eastern European populations are well suited to study the role of binge drinking in hypertension. We have therefore used data from a large population-based study in three Eastern European countries to investigate the following questions. First, whether both drinking volume/frequency and binge drinking are associated with high BP after controlling for both drinking volume and frequency (independent effect). Second, whether the effects of a given volume of alcohol consumption is stronger in the presence of binge drinking (effect modification). Finally, we also examined whether different alcoholic beverages have differential effect on the risk of high BP.

## Methods

The study was approved by ethical committees at University College London (UK), the Jagiellonian University, Krakow (Poland), the National Institute of Public Health, Prague (Czech Republic) and the Institute of Internal Medicine, Novosibirsk (Russia); all participants gave written consent.

### Study populations and subjects

We used cross-sectional data from the baseline survey for the Health, Alcohol and Psychosocial factors In Eastern Europe (HAPIEE) study, conducted in 2003–2005. Details on the study design and procedures have been reported elsewhere [25]. The study recruited men and women aged 45–69 years, randomly selected from electoral lists in Novosibirsk (Russia) and from population registers in Krakow (Poland) and 6 Czech towns. In total, the study recruited 28,947 persons (overall response rate 59%). From these, the following subjects were excluded: (i) 3,089 persons who did not attend the clinic for blood pressure measurement; (ii) 1,596 Polish participants interviewed by 5 nurses whose data failed quality control for alcohol consumption; (iii) 1,329 persons who refused to answer questions on alcohol consumption; and (iv) 7,903 persons who reported taking antihypertensive medication in the last two weeks. The remaining 15,030 subjects were included in the analysis.

### Measurements

Blood pressure was measured by trained nurses according to standardized protocol, using Omron 5Mi sphygmomanometer. For each participant, three measurements in sitting position were recorded, the first after 5 minute rest and subsequent readings after 2 minute intervals. The means of the second and third measurements of systolic and diastolic BP were used in the analyses. Self-reported details on physician-diagnosed hypertension and its treatment were collected using standard health-related questionnaire. The binary variable of high blood pressure was defined as systolic BP≥140 and/or diastolic BP≥/90 mm Hg. Persons reporting treatment by antihypertensive medication were excluded.

Alcohol consumption in the last year was ascertained by interviewer administered structured questionnaire. The reliability of the responses was assessed by investigating the proportion of abstainers and distribution of annual ethanol intake between observers, and data collected by interviewers with unusually high rates of underreporting were excluded from the analysis.

The main alcohol indices analysed here were derived from the graduated frequency questionnaire (GFQ) [26], containing nine mutually exclusive categories of frequency (ranging from “never” to “daily/almost daily”) and six mutually exclusive categories of amounts of ethanol consumed per single occasion, expressed in local units (0.5 l of beer, 0.2 l of wine and 0.05 l of spirits), ranging from “10 and above” to “less than one”. Participants were asked to convert different beverages into the units above and report the total alcohol units per occasion. Alcopops were not included, since their consumption by older persons in Eastern Europe is extremely rare. Total annual consumption of alcohol was calculated from drinking frequency and amounts consumed; 100 ml of beer, wine and spirit was assumed to contain 4 g, 10 g, or 36 g of ethanol, respectively. Persons were classified into 5 categories of drinking frequency and into 5 categories of annual alcohol intake (with lower cut-off points of annual intake for women than for men, see tables for detail).

In addition to assessing annual intake and drinking frequency, the GFQ data were also used to estimate the prevalence binge drinking. Binge drinking was defined as drinking 100 g (men) or 60 g (women) of ethanol in one session at least once a month. This cut off point in males is slightly higher than the 80 g [27] or 5 drinks [12] thresholds used in most studies.

Finally, we also categorized drinkers by beverage preference. The type of alcoholic beverage predominantly consumed was assessed using separate standard questions on consumption of particular types of alcoholic beverage during a typical week. Persons were classified into particular categories when the intake of ethanol of one type of beverage (beer, wine, or spirits) was at least 75% of the total ethanol consumed. Participants not meeting these criteria were assumed to not to have a preference.

Information on education and smoking was also collected by interviewer-administered questionnaire. For the purpose of this analysis we categorised education into 4 categories: primary or less, vocational, secondary, and university degree. We used 2 categories of smoking habit: non-smokers and current smokers. Participants' height and weight, measured without shoes and heavy outer garments using an electronic scale and mechanic stadiometer, were used to calculate body mass index (BMI, kg/m^2^).

### Statistical Analysis

Initial analyses were performed separately by country and sex. Because the associations between alcohol and blood pressure were similar across countries (none of the interactions between alcohol and country in either sex were statistically significant), data from all countries were pooled. Since the results differed by sex, data on men and women were analysed separately.

We used logistic and linear regression to assess the association between the prevalence of hypertension, and mean systolic and diastolic blood pressure, respectively, and alcohol. For each outcome, for each sex separately, we fitted three sets of models. First, we adjusted for age and country. Second, we also added education, smoking and body mass index. Finally, we have additionally included measures of annual alcohol intake and binge drinking simultaneously. Where appropriate, we also tested for linear trends by using the categorical independent variables as continuous variables.

Interactions between annual intake of alcohol and binge drinking (and, separately, by beverage preference) were tested in stratified analyses and by regression models comparing likelihood ratios of models with and without interaction terms; we did this both including and excluding non-drinkers (because there were, obviously, no binge drinkers among non-drinkers). Analyses were conducted using the SAS and Stata software.

## Results

The characteristics of persons included in the analysis by country and sex are shown in table 1. The prevalence of high blood pressure was highest in the Czech Republic among men and in Russia among women, and in each country males had higher blood pressure than females. Mean BMI was similar across countries. University education was less common among Czechs than among Russians and Poles, while current smoking in men was highest and in women lowest in Russia.

**Table 1:** Descriptive statistics of persons included in the analyses (persons using antihypertensive treatment were excluded).

	Czech Republic		Russia		Poland	
	Men n = 2050	Women n = 2452	Men n = 3321	Women n = 3030	Men n = 2188	Women n = 1989
Age (mean, SD)	56.8 (7.2)	55.8 (7.0)	57.3 (7.1)	56.5 (7.2)	56.3 (7.1)	55.0 (6.7)
Education (%)						
Primary or less	5.1	15.7	11.1	8.5	8.3	9.3
Secondary	75.8	72.1	58.5	62.8	60.2	57.9
University	19.1	12.2	30.4	28.7	31.5	32.8
BMI (mean, SD)	27.5 (3.6)	26.9 (4.5)	26.1 (4.2)	28.8 (5.3)	27.1 (3.8)	27.0 (4.5)
Smoking (%)						
Current	31.9	26.3	53.6	12.2	40.4	31.7
Past	35	20.4	22	4.8	32.3	22.8
Never	33.1	53.3	24.4	83	27.3	45.5
High BP (%)	57.8	36	52.9	44.3	47.7	27.1
Systolic BP (mmHg), mean (SD)	141.2 (17.8)	130.0 (18.1)	139.5 (21.8)	135.7 (23.4)	138.4 (19.2)	127.1 (17.7)
Diastolic BP (mmHg), mean (SD)	89.6 (10.6)	85.1 (10.4)	88.7 (12.8)	86.4 (12.6)	86.4 (11.2)	81.4 (10.3)
Drinking frequency (%)						
Never in last year	5.3	13.4	12.7	14.7	6.6	18.2
<1/month	15.8	33.1	16.6	55.3	23.1	39.9
1–3/month	16.7	25.3	24.5	21	27.3	26.2
1–4/week	38.8	23.1	40.1	8.7	33.9	14.1
5+/week	23.4	5.1	5.2	0.4	9.1	1.7
Annual alcohol intake (g), median, (IQR^1^)	3630 (821–9125)	600 (140–1950)	2990 (990–7132)	240 (100–660)	1530 (360–4080)	300 (90–830)
Mean alcohol dose per occasion (g), median, IQR^1^)	25 (18–45)	18 (14–25)	56 (34–79)	25 (18–25)	25 (15–38)	15 (10–25)
Binge drinking^2^ (%)	18.1	11.1	32	7.3	11.7	4.5

1 IQR: inter-quartile range.

2 Binge drinking: at least 100 g (men) or 60 g (women) of alcohol at least once a month.

There were considerable differences in the volume of alcohol consumption and style of drinking between the centres (table 1). The proportion of abstainers was lowest in Czech men and women and highest among Russian men and Polish women. Czech men and women had the highest median of the annual consumption of ethanol, largely because of the high frequency of drinking in the Czech Republic. However, these figures hide differences in the drinking pattern among males – in contrast to the annual intake, the mean dose of alcohol per drinking occasion, and the prevalence of binge drinking, were considerably higher in Russian men (56 g per occasion and 32% binge drinking) than in Czech and Polish men (25 g, 18% and 25 g, 12%, respectively).

### Relation between alcohol consumption and prevalence of hypertension

Despite differences in drinking pattern between countries, the relations between ethanol consumption indices and hypertension were similar across the countries. For example, the odds ratios of high BP for men consuming ≥12 litres of ethanol per year, compared to non-drinkers, were 2.35 (95% CI 1.51–3.67) in the Czech Republic, 2.31 (1.73–3.06) in Russia and 3.14 (1.73–5.10) in Poland (p for heterogeneity 0.468, not shown in table). Data from the 3 countries were therefore pooled (tables 2 and 3). In men, the odds of high BP increased with both annual intake and with drinking frequency. Further adjustment for BMI, smoking and education did not materially change the results. Linear regression analyses of systolic and diastolic BP yielded similar pattern of results: there were strong associations of BP with annual intake, drinking frequency and binge drinking.

**Table 2:** Numbers of subjects, odds ratios (95% confidence intervals) for high blood pressure and differences in means (standard errors) of systolic and diastolic blood pressure (mmHg) by drinking categories in men (n = 7559).

		High blood pressure		Systolic BP		Diastolic BP	
	N	OR (95% CI)^1^	OR (95% CI)^2^	Mean diff. (SE)^1^ mmHg	Mean diff. (SE)^2^ mmHg	Mean diff. (SE)^1^ mmHg	Mean diff. (SE)^2^ mmHg
Annual alcohol intake							
None	672	1.0 (reference)	1.0 (reference)	0 (reference)	0 (reference)	0 (reference)	0 (reference)
<3 L	1382	1.38 (1.16–1.63)	1.46 (1.22–1.75)	3.3 (0.8)	3.7 (0.8)	2.6 (0.5)	2.7 (0.5)
3–5.99 L	1757	1.83 (1.50–2.23)	1.94 (1.58–1.38)	5.0 (1.0)	5.2 (0.9)	4.0 (0.6)	3.9 (0.6)
6–11.99L	2897	1.93 (1.59–2.35)	2.06 (1.68–2.53)	5.8 (0.9)	6.0 (0.9)	4.6 (0.6)	4.6 (0.5)
12L+	851	2.54 (2.06–3.14)	2.73 (2.20–3.40)	9.9 (1.0)	10.0 (1.0)	6.9 (0.6)	6.9 (0.6)
P for trend		<0.001	<0.001	<0.001	<0.001	<0.001	<0.001
Drinking frequency							
Never	672	1.0 (reference)	1.0 (reference)	0 (reference)	0 (reference)	0 (reference)	0 (reference)
<1/mo	3794	1.33 (1.10–1.64)	1.41 (1.16–1.72)	3.0 (0.9)	3.4 (0.9)	2.3 (0.6)	2.3 (0.5)
1–3/mo	1087	1.54 (1.29–1.85)	1.60 (1.32–1.93)	4.3 (0.9)	4.4 (0.9)	3.3 (0.5)	3.2 (0.5)
1–4/w	1133	1.78 (1.50–2.11)	1.92 (1.60–2.29)	5.3 (0.8)	5.7 (0.8)	4.3 (0.5)	4.3 (0.5)
5+/w	873	2.14 (1.73–2.65)	2.39 (1.92–2.99)	7.5 (1.0)	8.1 (1.0)	5.2 (0.6)	5.5 (0.5)
P for trend		<0.001	<0.001	<0.001	<0.001	<0.001	<0.001

1 adjusted for age and country.

2 adjusted for age, country, body mass index, smoking and education.

**Table 3:** Numbers of subjects, odds ratios (95% confidence intervals) for high blood pressure and differences in means (standard errors) of systolic and diastolic blood pressure (mmHg) by drinking categories in women (n = 7471).

		High blood pressure		Systolic BP		Diastolic BP	
	N	OR (95% CI)^1^	OR (95% CI)^2^	Mean diff. (SE)^1^ mmHg	Mean diff. (SE)^2^ mmHg	Mean diff. (SE)^1^ mmHg	Mean diff. (SE)^2^ mmHg
Annual alcohol intake							
None	1134	1.0 (reference)	1.0 (reference)	0 (reference)	0 (reference)	0 (reference)	0 (reference)
<1 L	4788	1.20 (1.04–1.38)	1.27 (1.10–1.47)	1.3 (0.6)	1.9 (0.6)	1.0 (0.4)	1.3 (0.4)
1–2.99 L	982	1.13 (0.93–1.36)	1.23 (1.01–1.50)	1.6 (0.9)	2.6 (0.8)	1.6 (0.5)	2.1 (0.5)
3–5.99 L	318	1.38 (1.06–1.81)	1.63 (1.23–2.15)	1.5 (1.2)	3.2 (1.2)	1.4 (0.7)	2.4 (0.7)
6L+	249	1.14 (0.84–1.55)	1.35 (0.98–1.84)	2.6 (1.4)	4.2 (1.3)	1.7 (0.8)	2.7 (0.8)
P for trend		0.145	0.005	0.046	<0.001	0.003	<0.001
Drinking frequency							
Never	1134	1.0 (reference)	1.0 (reference)	0 (reference)	0 (reference)	0 (reference)	0 (reference)
<1/mo	3279	1.24 (1.07–1.44)	1.29 (1.11–1.50)	1.6 (0.7)	2.0 (0.7)	1.1 (0.4)	1.3 (0.4)
1–3/mo	1178	1.11 (0.95–1.51)	1.21 (1.02–1.43)	1.2 (0.7)	2.2 (0.7)	1.2 (0.4)	1.6 (0.4)
1–4/w	1108	1.23 (1.02–1.47)	1.37 (1.14–1.66)	1.6 (0.8)	2.9 (0.8)	1.4 (0.5)	2.0 (0.5)
5+/w	172	0.96 (0.67–1.37)	1.23 (0.85–1.79)	−0.7 (1.6)	2.0 (1.5)	0.4 (0.9)	2.1 (0.9)
P for trend		0.493	0.022	0.418	0.003	0.042	<0.001

1 adjusted for age and country.

2 adjusted for age, country, body mass index, smoking and education.

In women, the associations were generally weaker than in men (table 3). Age- and country-adjusted associations were inconsistent, only after further adjustment became the trends in BP by annual intake and drinking frequency apparent.

### Drinking pattern and hypertension

Table 4 examines whether binge drinking affects blood pressure independently from conventional indices of alcohol intake, by additionally adjusting the effects of binge drinking for annual intake. In men, BP was significantly higher in binge drinkers but additional control for annual consumption considerably attenuated the effects of binge drinking; e.g., the odds ratio were reduced from 1.62 (1.45–1.82) to 1.20 (95%CI 1.03–1.39). By contrast, the inclusion of binge drinking in the model did not materially change the odds ratios for annual intake or drinking frequency (not shown in table). In women, we found little attenuation of effects of binge drinking by additional adjustment for annual intake; similarly to men, controlling for binge drinking did not change the estimated effects of annual intake and drinking frequency (not shown).

**Table 4:** Odds ratios (95% confidence intervals) of high blood pressure and differences (standard errors) in systolic and diastolic blood pressure (mmHg) by binge drinking (compared with non-bingers).

	High BP OR (95%CI)	Systolic BP Diff. (SE) mmHg	Diastolic BP Diff. (SE) mmHg
*Men*			
Model 1	1.62 (1.45–1.82)	5.0 (0.6)	3.5 (0.3)
Model 2	1.57 (1.40–1.78)	4.4 (0.5)	3.3 (0.3)
Model 3	1.20 (1.03–1.39)	1.9 (0.7)	1.6 (0.4)
*Women*			
Model 1	1.33 (1.11–1.59)	3.5 (0.8)	2.5 (0.5)
Model 2	1.35 (1.12–1.62)	3.6 (0.8)	2.5 (0.5)
Model 3	1.31 (1.05–1.63)	3.3 (1.0)	2.3 (0.6)

Model 1: adjusted for age and country.

Model 2: adjusted for age, country, body mass index, smoking and education.

Model 3: adjusted for age, country, body mass index, smoking, education and annual alcohol intake.

**Figure 1:**
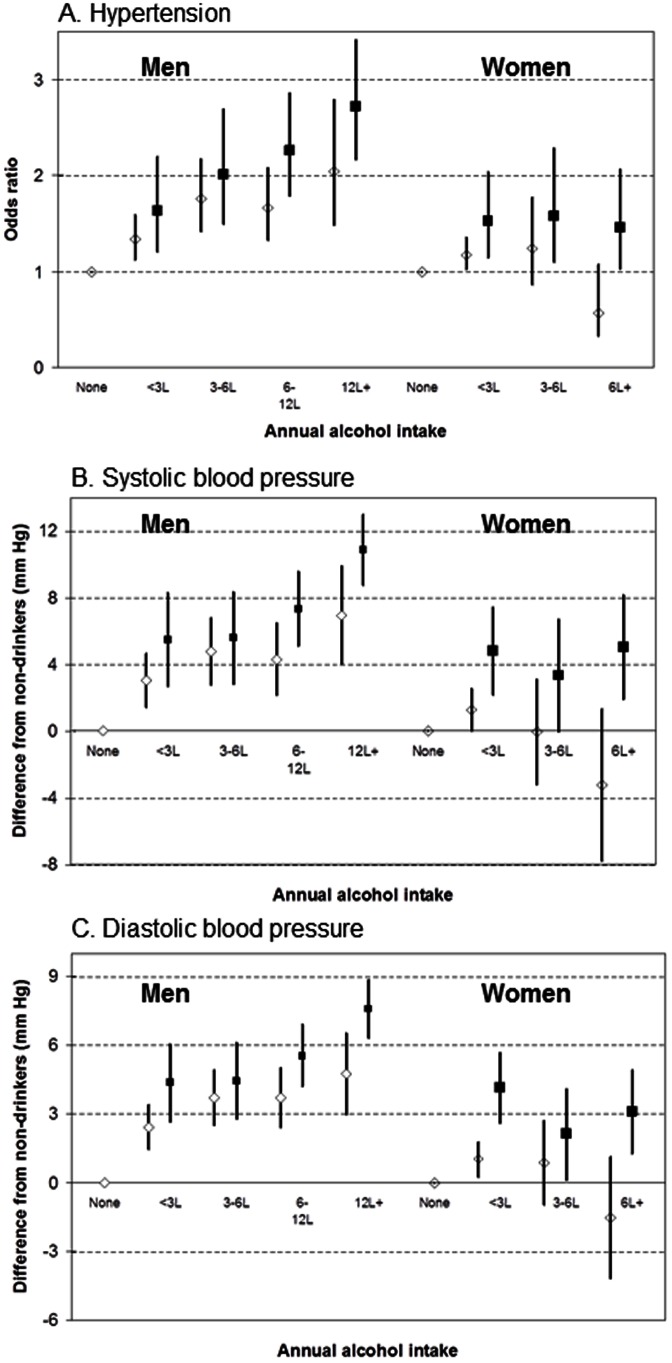
Odds ratios of hypertension (graph A) and differences in systolic (graph B) and diastolic (graph C) blood pressure, compared with non-drinkers, by annual alcohol intake and binge drinking, with 95% confidence intervals, adjusted for age and country, men and women separately.

Next, we have examined the hypothesis that binge drinking modifies the effect of annual intake (Figure 1). In men, the prevalence of high BP and the mean SBP and DBP increased with annual intake and they were, within each annual intake category, slightly higher among binge drinkers. In women, the association of annual intake with blood pressure was much weaker but, as in men, blood pressure was higher in binge drinkers than non-binge drinkers in each intake category. In neither sex were the interaction terms between annual alcohol intake and binge drinking statistically significant but, consistently with table 4, there were modest but statistically significant effects of binge drinking on blood pressure in both sexes.

An alternative analysis of drinking pattern, stratifying drinkers into binge and non-binge drinkers, is shown in table 5. If infrequent drinking of larger amounts (binge drinking) increases BP, then persons drinking the same amount of alcohol less frequently would have higher BP than those drinking more frequently. We found that, among men drinking ≥6 L of pure ethanol annually, after controlling for annual intake, levels of BP were marginally higher among infrequent drinkers. However, for none of the three outcomes was the difference statistically significant.

**Table 5:** Odds ratios (95% confidence intervals) for high blood pressure and differences in means (standard errors) of systolic and diastolic blood pressure (mm Hg) by drinking frequency among men consuming ≥6 litres of ethanol per year (n = 2006).

	High BP	Systolic BP	Diastolic BP
	OR (95% CI)	Mean diff. (SE) mmHg	Mean diff. (SE) mmHg
Drinking frequency			
5+/week	1	0 (reference)	0 (reference)
1–4/week	1.06 (0.84–1.32)	0.0 (1.0)	0.5 (0.6)
1–3/month	1.30 (0.80–2.11)	1.3 (2.2)	1.6 (1.3)
P for trend	0.359	0.742	0.21

Adjusted for age, country, body mass index, smoking, education and annual alcohol intake.

Beverage preference also did not modify the effect of drinking alcohol; after controlling for annual alcohol intake, the odds ratios of high BP (compared to non-drinkers) among drinkers preferring different drinks were very similar (table 6). Similarly, when beverage preference was added to the model with annual intake (and other covariates), none of beverage preferences were statistically significant (the smallest p-value was 0.23); none of the interaction between annual intake and beverage were statistically significant (not shown in table).

**Table 6:** Odds ratio for high BP in drinkers vs. non-drinkers of different beverages.

	Men		Women	
	OR^1^ (95%CI)	OR^2^ (95%CI)	OR^1^ (95%CI)	OR^2^ (95%CI)
Alcoholic beverage preference (drinkers vs. non-drinkers)^3^				
Beer	1.81 (1.51–2.18)	1.60 (1.31–1.95)	1.25 (1.02–1.54)	1.38 (1.11–1.72)
Wine	1.77 (1.30–2.42)	1.86 (1.34–2.57)	1.19 (0.96–1.48)	1.37 (1.09–1.73)
Liquor	1.76 (1.45–2.15)	1.53 (1.23–1.89)	1.35 (1.03–1.78)	1.41 (1.06–1.88)
No preference	1.84 (1.52–2.22)	1.64 (1.33–2.01)	1.34 (1.06–1.68)	1.35 (1.21–1.99)

1 adjusted for age and country.

2 adjusted for age, country, body mass index, education, smoking, and annual alcohol consumption.

3 reference category: non-drinkers.

## Discussion

This study, in large Central and East European population samples with high levels of male binge drinking, found a small effect of binge drinking on blood pressure once the frequency and volume of drinking were taken into account, and there was no evidence that the effect of drinking is modified by binge drinking pattern. Our study confirms strong effects of conventional measures of alcohol consumption, such as the volume and frequency of drinking, and we found no evidence of a protective effect of moderate drinking.

Several limitations of the study need to be considered when interpreting the results. The main issue is the cross-sectional design which may complicate the temporality. It is possible that persons with high BP reduce their drinking, leading to underestimation of an underlying association. However, this bias seems unlikely in our study; when the analyses were restricted to persons who were not aware of having high blood pressure, the results were virtually identical (not shown in tables).

Second, persons taking antihypertensive medication were excluded in order to eliminate the confounding effect of the negative relation between undergoing treatment and the alcohol consumption. This may affect the generalizability of the results. However, in analyses of the full data, persons taking antihypertensive medication had lower alcohol consumption and lower blood pressure. When these subjects were included in the analyses, the associations between alcohol and blood pressure, after adjusting for medication, were essentially the same as the main results. Similarly, inclusion of data collected by 5 Krakow nurses, because they were deemed to be of low quality, did not affect the results.

Third, self-reported measures of alcohol consumption are prone to underreporting and misclassification. In fact, when using additional variables not included in the main analyses, we found some evidence for under-reporting: non-drinkers and infrequent drinkers in the graduate frequency questionnaire (the main measure in the paper) reported some drinking when responding to a questions on typical weekly intake and in a food frequency questionnaire administered on a separate occasion. This under-reporting can plausibly explain the relatively high odds ratios among infrequent drinkers in the main analyses. In addition, underreporting may be more pronounced in women in Russia [28], where female drinking is associated with social stigma, although the correlation of serum GGT with self-reported drinking in Russians in this study was similar in men and women [29]. Reporting bias may explain some of the inconsistent findings in women but it is unlikely to play a major role in males. The fact that the GFQ demanded participants to combine different beverages into alcohol units introduced further complexity which may have led to further misclassification of alcohol consumption.

On the other hand, binge drinking, as a more visible way of drinking, may be less prone to misclassification than annual intake. If this is the case, the effect of binge drinking is underestimated to a lesser degree than the effect of volume or frequency of drinking; and this differential degree of misclassification, effectively leading a residual confounding [30], may underlie the small “independent” effect of binge drinking on BP. It is unlikely that the results were influenced by the way we classified alcohol consumption; we conducted a number of alternative analyses (not shown in tables) and the results were entirely consistent.

Third, the lack of the effect of binge drinking may be due to the interval since last drinking occasion. While we had no data on the timing of the last drinking session, day of the week has been found to be associated with drinking on other studies [15], [31], [32]; however, adjustment for day of the week did not alter the results.

Fourth, our measures of alcohol consumption covered the last year, and this classification may be affected by stopping or reducing drinking before the last 12 months. A question on stopping/reducing drinking in the past was available on our Russian sample, and stratifying the analyses by past consumption did not change the results. It is therefore unlikely that past drinking confounded our findings.

Fifth, the study assessed long-term effects of binge drinking but it was unable to capture short-term effects. It has been suggested the some of the effect of ethanol on blood pressure are short term [16]–[20], [33]. For example, a study in Northern Ireland (with predominantly weekend binge drinking pattern) found raised blood pressure on Mondays and Tuesdays, possibly reflecting weekend drinking, while no such effect was observed in France [15]. Given our definition of binge drinking at least once a month, our study may have therefore missed short term effects of binge drinking.

Finally, the response rate, although similar to many recent population studies, was relatively low. In epidemiological studies, non-responders tend to have higher levels of unhealthy behaviours and be less healthy (e.g. in Krakow, where follow up of non-respondents was possible, non-participants had higher mortality than participants). It is therefore possible that the levels of alcohol consumption and hypertension in the study samples were underestimated. However, it is unlikely that participation rate would introduce major bias in the estimates of the association between alcohol and blood pressure.

This study also had a number of strengths. Perhaps the most important is the choice of study populations. Eastern European countries have a high alcohol consumption and, particularly in the countries of the former Soviet Union, high rates of binge drinking [34]. In this study, one third of Russians reported drinking at least 100 g of ethanol at least once a month; this is consistent with earlier reports from Russia [23], [24], with high estimates of mortality attributable to alcohol in Eastern Europe [35], and with the inclusion of Russia and other post-Soviet republics into a group of countries with the most hazardous drinking pattern in the WHO Global Burden of Disease project [36]. Second, we used the graduated frequency questionnaire, which is considered to be the most suitable tool to assess binge drinking [26]. Third, the large sample size provides the study with sufficient statistical power, although among women the numbers of binge/heavy drinkers were low, perhaps partly due to the under-reporting in women mentioned above.

With respect to the conventional measures of alcohol consumption, our results confirm previous findings that alcohol intake and drinking frequency are associated with high blood pressure in an approximately linear fashion [2]–[8]. with the magnitude of the effects observed in this study in the upper end of the range of results reported previously. Consistent with the majority of previous studies, we found no evidence of a threshold or protective effect of alcohol. Some of the inconsistent results in women may be due to misreporting of alcohol intake, although some previous studies have suggested weaker effects of alcohol on BP in women than in men [6], [37].

Binge drinking was the main focus of this study, because it has been previously linked with increased risk of hypertension and cardiovascular risk in general. We found a relatively modest effect of binge drinking on blood pressure after controlling for the volume of drinking. We used several alternative ways to analyse the possible interaction between alcohol intake and binge drinking but our data do not support the hypothesis that drinking pattern modified the effect of alcohol intake. The fact that associations between alcohol and blood pressure were homogenous across participating countries also contradicts the hypothesis that drinking pattern modifies the effect of ethanol – given the high prevalence of binge drinking in Russia, one would expect stronger effects of a given alcohol intake on blood pressure in Russia; this was not the case.

Current evidence on this subject is surprisingly sparse. The largest population-based study to date, the PRIME study, provided indirect evidence based on ecological comparison of day of week variation in BP between Northern Ireland and France [15]. However, population-based studies using individual data did not find significantly increased rates of hypertension in weekend-only drinkers (analogous to binge drinkers) [5], in binge drinkers [19] or in persons drinking similar amounts over fewer occasions [38]. A recent Danish study of genetic variants linked with alcohol consumption found no relationship between the ALDH2 genotype (the only genotype associated with binge drinking) and blood pressure [39]. Some of the earlier smaller studies either examined very short-term effects of a single drinking occasion [17] or did not find differences between binge and regular drinkers [16], [18]. Only Abramson and colleagues found higher levels of ambulatory BP in binge drinkers after controlling for weekly drinking volume [20]. Overall, our finding of only a modest effect of binge drinking seems in line with the balance of the existing literature.

Several studies reported a positive association between blood pressure and drinking without meals, another dimension of drinking pattern [5], [40]. Anecdotally, drinking without meals is common in Russia but we had no data to address this issue. We were, however, able to assess the effect of preferred alcoholic beverage. There have been reports that different alcoholic beverages have differential cardiovascular health effects, with wine often presented as the “healthiest” [22]. We found no evidence to support these claims, and our negative results are consistent with earlier population-based studies [3], [5], [38].

Our findings may have implications for both public health and medical practice. Firstly, although moderate alcohol consumption is associated with slightly lower risk of coronary heart disease, our (and others') results demonstrate a linear dose-response association between ethanol and blood pressure; this should lead to more cautious recommended levels of alcohol consumption. Secondly, while binge drinking has a number of negative consequences on health, the evidence that it has a clinically meaningful long term effect on blood pressure, once total alcohol intake is taken into account, is not supported by our results. And finally, many people view wine as more healthy type of alcohol but our results corroborate earlier findings that blood pressure is increased by all types of alcoholic beverages to a similar extent.
